# Zinc Protoporphyrin Suppresses β-Catenin Protein Expression in Human Cancer Cells: The Potential Involvement of Lysosome-Mediated Degradation

**DOI:** 10.1371/journal.pone.0127413

**Published:** 2015-05-22

**Authors:** Shuai Wang, Bethany N. Hannafon, Stuart E. Lind, Wei-Qun Ding

**Affiliations:** 1 Department of Pathology, University of Oklahoma Health Sciences Center, Oklahoma City, Oklahoma, United States of America; 2 Departments of Pathology and Medicine, University of Colorado School of Medicine, Aurora, Colorado, United States of America; University of South Alabama, UNITED STATES

## Abstract

Zinc protoporphyrin (ZnPP) has been found to have anticancer activity both *in vitro* and *in vivo*. We have recently demonstrated that ZnPP diminishes β-catenin protein expression in cancer cells. The present study examined the cellular mechanisms that mediate ZnPP’s suppression of β-catenin expression. We demonstrate that ZnPP induces a rapid degradation of the β-catenin protein in cancer cells, which is accompanied by a significant inhibition of proteasome activity, suggesting that proteasome degradation does not directly account for the suppression. The possibility that ZnPP induces β-catenin exportation was rejected by the observation that there was no detectable β-catenin protein in the conditioned medium after ZnPP treatment of cancer cells. Further experimentation demonstrated that ZnPP induces lysosome membrane permeabilization, which was reversed by pretreatment with a protein transportation inhibitor cocktail containing Brefeldin A (BFA) and Monensin. More significantly, pretreatment of cancer cells with BFA and Monensin attenuated the ZnPP-induced suppression of β-catenin expression in a concentration- and time-dependent manner, indicating that the lysosome protein degradation pathway is likely involved in the ZnPP-induced suppression of β-catenin expression. Whether there is cross-talk between the ubiquitin-proteasome system and the lysosome pathway that may account for ZnPP-induced β-catenin protein degradation is currently unknown. These findings provide a novel mechanism of ZnPP’s anticancer action and reveal a potential new strategy for targeting the β-catenin Wnt signaling pathway for cancer therapy.

## Introduction

Zinc protoporphyrin (ZnPP) belongs to a group of chemical compounds in which the central ion of free heme is replaced by a heavy metal ion, such as zinc, tin (SnPP) or copper (CuPP). Due to ZnPP’s structural similarity to that of free heme, an established substrate of the antioxidant enzyme heme oxygenase-1 (HO-1), ZnPP acts as a competitive inhibitor for HO-1 enzymatic activity [1]. ZnPP has been found to have anticancer activity both *in vitro* and *in vivo* [2–5] and it is generally believed that ZnPP’s anticancer activity is attributed to HO-1 inhibition. However, experimental evidence has not been provided to support this assumption. On the contrary, a few studies have suggested that ZnPP’s anticancer action might be independent of HO-1 [5,6]. In our recent report, we demonstrated that neither over-expression nor knockdown of HO-1 in cancer model systems affects ZnPP’s cytotoxicity, strongly indicating an HO-1-independent action of ZnPP against cancer cells. Our mechanistic studies further revealed that ZnPP is able to rapidly and dramatically suppress β-catenin protein expression and activity in cancer cells [7].

Because β-catenin is a key player in the canonical Wnt signaling pathway, which is a well appreciated target pathway for cancer therapy [8], the significant suppression of β-catenin expression and activity reveals an important mechanism of ZnPP’s anticancer activity. A further understanding of how ZnPP suppresses β-catenin expression in cancer cells may not only help elucidate the cellular mechanisms of ZnPP’s anticancer action, but also provide new cancer therapeutic strategies for targeting the β-catenin Wnt signaling pathway.

In the present study, we have explored the cellular mechanisms of ZnPP-induced suppression of β-catenin expression in human cancer cells. The rapid and dramatic nature of the ZnPP-induced suppression of β-catenin protein expression strongly suggests that this suppression is primarily due to β-catenin protein degradation. β-catenin protein levels are well controlled by the β-catenin destruction complex that is tightly coupled to the ubiquitin-proteasome system [9]. It is therefore likely that the ubiquitin-proteasome system mediates ZnPP-induced β-catenin protein degradation. However, other protein degradation pathways, such as the lysosome-mediated protein degradation pathway [10], may also be involved in this process. In addition, the possibility that ZnPP induces rapid exportation of β-catenin from cancer cells cannot be excluded. The present study examined these three potential mechanisms of ZnPP-induced suppression of β-catenin expression. To our surprise, ZnPP-induced suppression of β-catenin expression is not due to enhanced proteasome activity nor is it mediated by exportation of β-catenin. Our results support the involvement of the lysosome-mediated degradation pathway in the ZnPP-induced suppression of β-catenin expression.

## Material and Methods

### Materials

The β-catenin, phospho-β-catenin (Ser33/37/Thr41) and K48 (lysine 48)-linkage specific polyubiquitin antibodies were from Cell Signaling Technology, Inc. (Danvers, MA). MG132 and Brefeldin A/Monensin cocktail were from Cayman Chemical (Ann Arbor, MI). Suc-LLVY-AMC was from Anaspec (Fremont, CA). Z-ARR-AMC and Z-LLE-AMC were from Millipore (Billerica, MA). Other fluorescent probes were from Life Technologies (Grand Island, NY). The Corning Spin-X concentrators (6 mL) and monensin sodium salt was from VWR International LLC (Radnor, PA). The β-actin antibody and other chemical reagents were analytic grade and obtained from Sigma-Aldrich (St. Louis, MO).

### Cell culture

The A2780 cell line (human ovarian cancer) was a kind gift from Dr. Stephen Howell (University of California, San Diego). The DU145 cell line (human prostate cancer) and MDA-MB-231 cell line (human breast cancer) were purchased from American Type Culture Collection (ATCC, Manassas, VA). A2780 cells were cultivated in RPMI 1640 medium, and DU145 and MDA-MB-231 cells were cultivated in DMEM medium. Both RPMI 1640 and DMEM mediums were supplemented with 10% fetal bovine serum, 100 IU/ml penicillin, and 100 μg/ml streptomycin. Cells were routinely grown in a 75-mm flask at 37°C in a humidified environment containing 5% CO_2_. All cells were sub-cultivated twice a week and applied to the various experiments as described in the results section.

### Preparation and application of ZnPP and SnPP

ZnPP and SnPP were purchased from Frontier Scientific, Inc. (Logan, UT). The manufacturer’s advice and a previous report [11] were followed for proper handling of these compounds. A working stock of ZnPP and SnPP was freshly prepared for each individual experiment. All tubes used to prepare the stock solution were covered by aluminum foil to avoid light reaction with the compounds. The compounds were initially dissolved in complete DMSO, and further diluted with 50% DMSO in 1X PBS buffer prior to addition to the cell culture medium. The final DMSO concentration in the cell culture medium was below 0.5% in all experiments conducted. Vehicles were included as controls. Cells were treated with the compounds in indirect low-light conditions and incubated in the dark for various lengths of time prior to individual assays, similar to previous reports [5,11].

### Western blot analysis

Protein expression was analyzed by Western blot as we previously described [12,13]. Cells were seeded into 100-mm culture dishes and reached 80% confluence prior to the treatment with ZnPP or SnPP at indicated concentrations and durations. For whole cell lysate, cells were lysed and sonicated on ice for 3 strokes (10 seconds each with 10 seconds interval in between). Insoluble materials were removed by centrifugation at 15,000×g for 15 min. The supernatants were collected for protein concentration determination. For extracellular protein isolation, cells were treated in Hanks’ balanced salt solution (HBSS) for 1.5 hours. After treatment, HBSS was collected and concentrated with Corning Spin-X concentrators. 25 to 40 μg of protein were loaded onto each well of a 10% SDS PAGE gel, transferred to a PVDF membrane, and blotted with specific antibodies against β-catenin, phosphorylated β-catenin, polyubiquitinated proteins and β-actin.

### Co-immunoprecipitation

Co-immunoprecipitation (co-IP) using β-catenin antibody was performed as described previously [13]. In short, cells growing in 100-mm dishes were washed with PBS and harvested by adding 150 μl of IP buffer (10 mM Tris-HCl, pH 7.4, 50 mM NaCl, 0.5 mM EDTA, 1 mM PMSF, and 1% Triton X-100). The cells were sonicated for 1 minute on ice, and insoluble material was removed by centrifugation. Supernatants were collected and protein concentrations determined. Supernatants were pre-cleared by agarose-coupled protein A, and the agarose beads were removed by centrifugation. β-catenin antibodies were added to the supernatants (1:100 ratio), and the reaction was incubated at 4°C overnight with gentle rotation. 50 μl of agarose-coupled protein A was added to capture the antibody-protein complexes by rotating for 2 hours at 4°C. The beads (IPs) were then collected by centrifuging at 2,000 × g for 5 minutes. The IPs were Solubilized with 50 μl of 2 X SDS-PAGE buffer by mixing and incubating for 1 hour at room temperature. Supernatant was collected by centrifugation at 2,000 × g for 5 minutes. A parallel immunoprecipitation with rabbit IgG was performed as a control. Both the IPs and inputs were boiled for 5 minutes and western blot was performed using antibodies against K-48 linkage specific polyubiquitinated proteins and β-catenin. β-actin expression in the inputs were was also determined.

### Fluorescence microscopic detection of intracellular ZnPP and lysosome permeability

Lysosome permeability was analyzed by fluorescence microscopy using the Operetta High Content Imaging System from PerkinElmer (Waltham, MA). A2780 cells were plated in Cell Carrier-96 plate from PerkinElmer (Waltham, MA) at a density of 10,000 cells per well. Forty-eight hours after plating, the cells were treated with ZnPP or SnPP or pre-treated with Brefeldin A/Monensin (21.1 μM / 4 μM) cocktail for 4 hours followed by treatment with ZnPP. The medium was then replaced with fresh medium containing 2.5 μM Acridine Orange (AO, Invitrogen, Carlsbad, CA). After 30 minutes incubation the cells were washed three times with HBSS and viewed under the Operetta. Lysosome permeability was measured using the AO staining [14]. AO was detected by excitation at 500 nm, emission at 526 nm for red, and excitation at 460 nm, emission at 650 nm for green.

### Proteasome activity assay

Proteasome activities were measured as previously reported [15]. In brief, A2780 cells were treated with ZnPP, SnPP or MG132 at different concentrations and durations. After treatment, cells were washed with PBS and collected in PBS. Cell pellets were lysed with 250 μl lysis buffer (50 mM HEPES, pH 7.5, 5 mM EDTA, 150 mM NaCl and 1% Triton X-100) per 5×10^6^ cells by incubating at room temperature for 30 minutes and vortexing every 10 minutes. The lysates were then centrifuged and supernatant collected. A total of 10 μg of protein for each sample was incubated with 20 µM fluorogenic substrate (Suc-LLVY-AMC, Z-ARR-AMC or Z-LLE-AMC) in 100 μl assay buffer (20 mM Tris-HCl, pH 7.5) at 37°C for 2 hours. After incubation, the fluorescence was read at 380 nm excitation and at 460 nm emission using Molecular Devices Fmax fluorescent microplate reader (Sunnyvale, CA)

## Results

**Fig 1:**
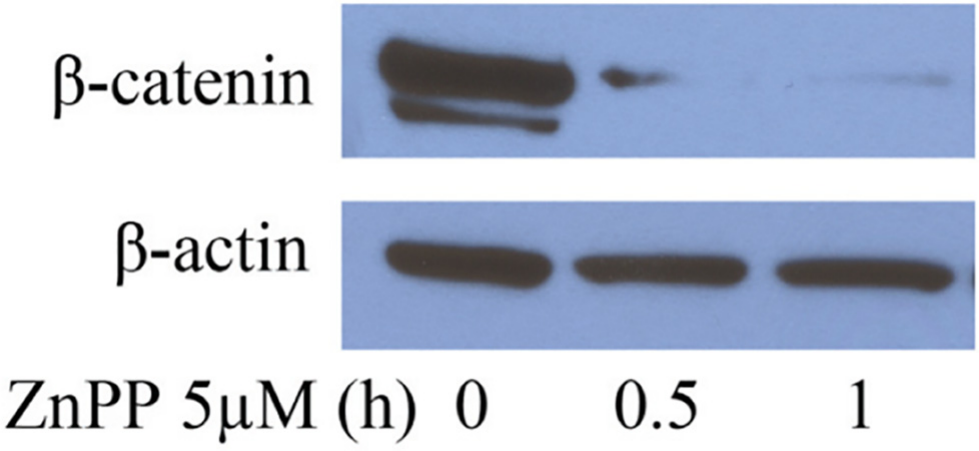
ZnPP suppresses β-catenin protein expression in A2780 cells. A2780 cells were treated with 5 μM ZnPP for 0.5 or 1 hour. Cell lysates were prepared and western blot was performed using antibodies against β-catenin and β-actin.

**Fig 2:**
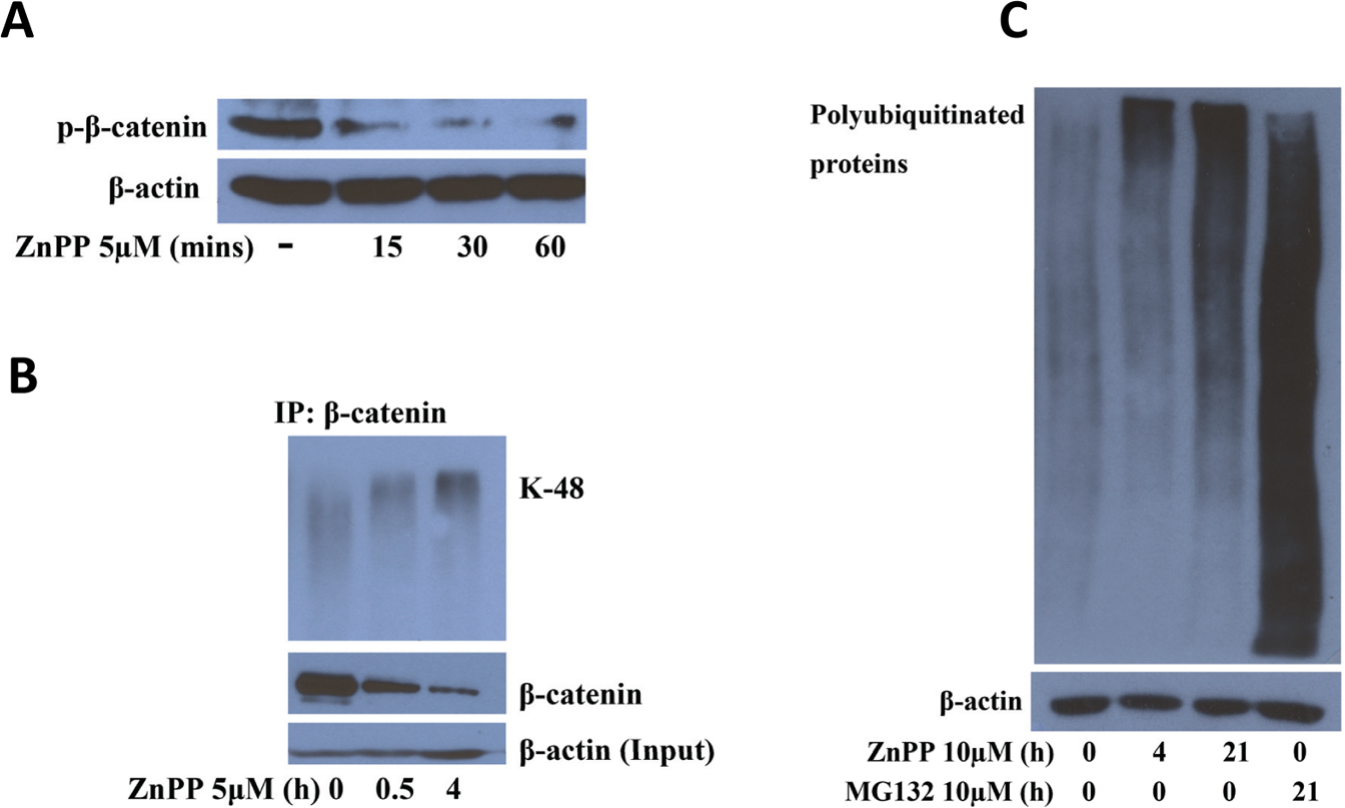
ZnPP induces accumulation of poly-ubiquitinated proteins. ***A*.** A2780 cells were treated with 5 μM ZnPP for 15, 30 or 60 minutes. Cell lysates were prepared and western blot was performed using antibodies against phosphorylated β-catenin (Ser 33, Ser 37 and Thr 41) and β-actin. ***B*.** A2780 cells were treated with 5 μM ZnPP for 0.5 and 4 hours. Cell lysates were prepared and co-IP was performed using β-catenin antibody followed by western blot analysis of k48 linkage specific proteins, β-catenin (IPs) or β-actin (inputs). Shown are representative images of three individual experiments. ***C*.** A2780 cells were treated with 10 μM ZnPP for 4 and 21 hours, or 10 μM MG132 for 21 hours. Cell lysates were prepared and western blot was performed using antibodies against K48-linkage specific polyubiquitinated proteins and β-actin.

The ZnPP-induced suppression of β-catenin expression is accompanied by an inhibition of proteasome activity. We have previously reported that ZnPP suppresses β-catenin protein expression in human cancer cells [7]. This was also confirmed in the present study (Fig 1). Treatment with 5 μM ZnPP for 30 minutes to 1 hour dramatically suppressed β-catenin protein expression in A2780 cells, indicating that protein degradation is involved. β-catenin protein levels are tightly regulated by the ubiquitin-proteasome system. In the absence of Wnt ligands, the β-catenin protein can be phosphorylated by CK1 at Ser 45, followed by a secondary phosphorylation at Ser 33, Ser 37, and Thr 41 by GSK-3β. Phosphorylated β-catenin will then be poly-ubiquitinated and targeted for degradation by the proteasome [9]. To determine whether activation of proteasome activity is the primary mechanism for ZnPP-induced β-catenin degradation, we measured the level of phosphorylated β-catenin after ZnPP treatment in A2780 cells. Fig 2A shows that the level of phosphorylated β-catenin (Ser 33, Ser 37 and Thr 41) was decreased after treatment with 5 μM ZnPP for 15, 30 or 60 minutes, indicating that ZnPP does not enhance phosphorylation of β-catenin by GSK-3β. Co-immunoprecipitation with an antibody against β-catenin (rabbit IgG used as control for precipitation) further showed that while the levels of whole β-catenin was suppressed, K48 linkage specific poly-ubiquitinated β-catenin accumulated in the β-catenin precipitated samples after ZnPP treatment for 30 minutes and 4 hours (Fig 2B). We then measured the levels of K48 (lysine 48)-linkage specific poly-ubiquitinated proteins to further determine the effects of ZnPP on poly-ubiquitinated proteins. The K48-linked poly-ubiquitin chain is known to target proteins for proteasomal degradation [16]. As shown in Fig 2C, treatment with 10 μM ZnPP for 4 or 21 hours induced accumulation of K48 specific poly-ubiquitinated proteins, indicating that rather than activating proteasome activity, ZnPP actually suppresses proteasome activity in our model system. Note that zinc binding compounds have previously been described to inhibit proteasome activity [17,18].

**Fig 3:**
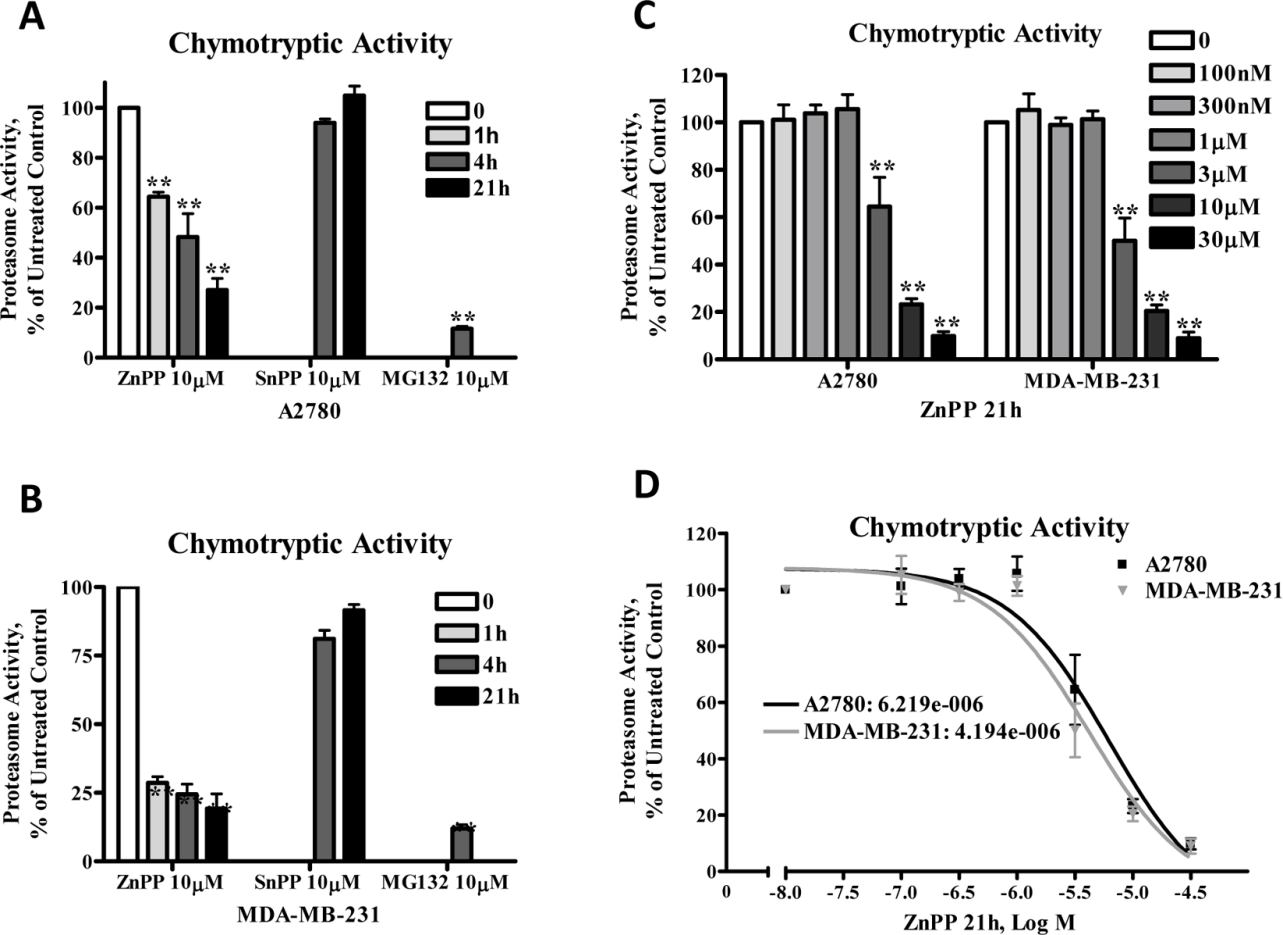
ZnPP inhibits chymotryptic proteasome activity in A2780 and MDA-MB-231 cells. A2780 ***(A)*** or MDA-MB-231 ***(B)*** cells were treated with 10 μM ZnPP, SnPP or MG132 for 4 or 21 hours. Whole cell lysates were prepared and incubated with Suc-LLVY-AMC for 2 hours at 37°C and fluorescence was recorded at 380 nm excitation and 460 nm emission. ***C*, *D*.** A2780 and MDA-MB-231 cells were treated with various concentrations of ZnPP as indicated for 21 hours. Whole cell lysates were prepared and incubated with Suc-LLVY-AMC for 2 hours at 37°C and fluorescence was recorded at 380 nm excitation and 460 nm emission. The IC_50_ was calculated with a nonlinear regression curve (Sigmoidal dose-response equation). Data (mean ± SE, n = 3) are expressed as percentages of untreated control. **, *P* < 0.01, compared to untreated control cells using one-way ANOVA followed by Bonferroni analysis.

**Fig 4:**
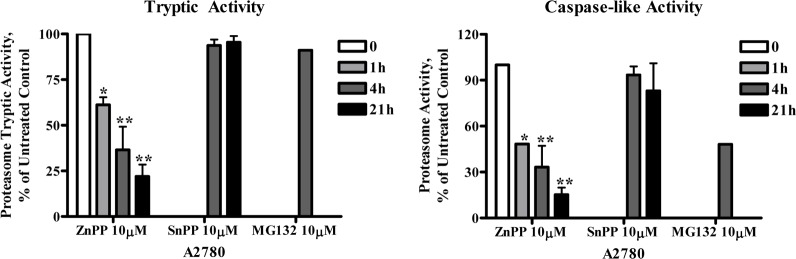
ZnPP inhibits tryptic and caspase-like proteasome activity. A2780 cells were treated with 10 μM ZnPP, SnPP or MG132 for 4 or 21 hours. Whole cell lysates were prepared and incubated with Z-ARR-AMC (tryptic activity) ***(A)*** or Z-LLE-AMC (chymotryptic activity) ***(B)*** for 2 hours at 37°C and fluorescence was recorded at 380 nm excitation and 460 nm emission. Data (mean ± SE, n = 3) are expressed as percentages of untreated control. *, *P* < 0.05, **, *P* < 0.01, compared to untreated controls using one-way ANOVA followed by Bonferroni analysis.

ZnPP’s inhibition of proteasome activity was further confirmed by direct measurement of the 20S proteasome chymotryptic activity, which was analyzed using the fluorophore linked peptide Suc-LLVY-AMC [19]. The eukaryotic 20S proteasome is known to have activities attributed to its various protein subunits that are referred to as caspase-like activity (cleaves after Glutamine and Aspartic acid residues), trypsin-like activity (cleaves after the basic amino acids Lysine and Arginine) and chymotrypsin-like activity (cleaves after hydrophobic amino acids) [20]. As shown in Fig 3, treatment of A2780 (Fig 3A and 3C) and MDA-MB-231 (Fig 3B and 3C) cells with ZnPP or MG132, but not SnPP, suppressed chymotryptic activity in a time- and concentration-dependent manner. The IC_50_ for ZnPP’s inhibition of proteasome activity was determined to be 6.2 μM in A2780 cells and 4.2 μM in MDA-MB-231 cells (Fig 3D). ZnPP, but not SnPP, also suppressed the tryptic and caspase-like proteasome activity in A2780 cells as analyzed using the fluorophore linked peptides Z-ARR-AMC or Z-LLE-AMC, respectively, [19] (Fig 4). Note that MG132 was more effective in suppressing the chymotryptic activity, rather than the caspase-like activity and did not affect the tryptic activity, results which are consistent with previous reports [21,22].

These results indicate that ZnPP-induced β-catenin protein degradation is accompanied by a significant suppression of ubiquitin-proteasome activity, suggesting that proteasome degradation does not directly account for ZnPP-induced suppression of β-catenin expression.

**Fig 5:**
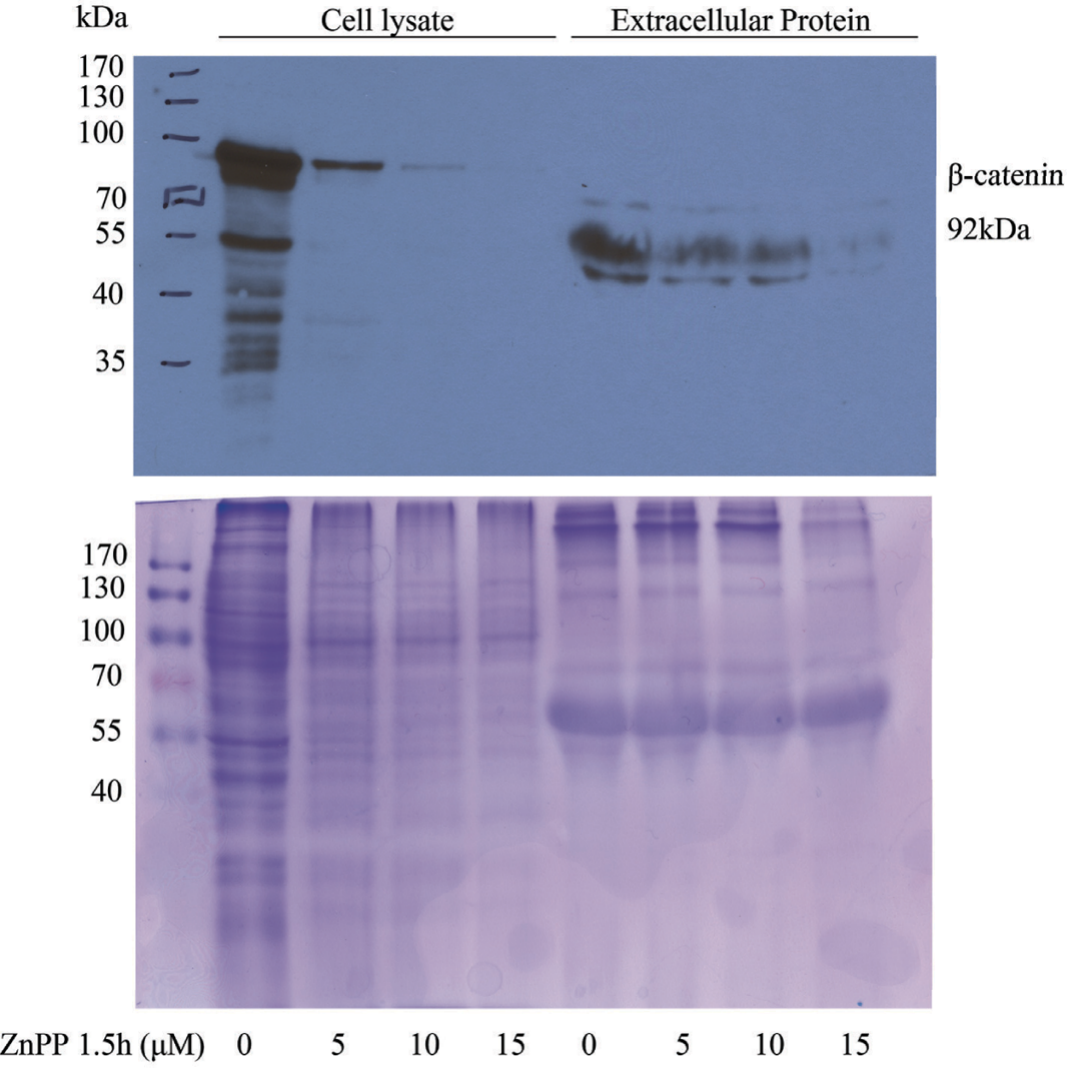
ZnPP does not induce secretion of β-catenin protein. A2780 cells were cultured in HBSS and treated with ZnPP at the indicated concentrations for 1.5 hours. The conditioned HBSS was collected and the proteins were concentrated. Western blot was performed using antibodies against β-catenin ***(Top)*.** Cell lysates and concentrated protein samples were also separated by SDS-PAGE and stained with Coomassie blue staining ***(Bottom)***. Shown are representative images of three individual experiments.

ZnPP does not promote β-catenin protein exportation. A recent report demonstrated that the β-catenin protein is secreted in exosomes from HEK293 cells, leading to a significant suppression of the canonical Wnt signaling pathway [23]. To determine whether ZnPP-induces β-catenin protein secretion, A2780 cells were cultured in HBSS and treated with 5 μM or 10 μM of ZnPP for 1.5 hours. Whole cell lysates and concentrated extracellular proteins were prepared. Approximately 20–30 μg of total cell lysate and extracellular proteins were loaded onto a SDS-polyacrylamide gel. Western blot analysis (Fig 5 upper) shows that β-catenin protein expression was suppressed by ZnPP in the cell lysate and undetectable in the extracellular protein fraction, indicating that ZnPP does not induce β-catenin protein secretion. Some low molecular bands were only detected in extracellular proteins, not in whole cell lysates, suggesting that these are non-specific. This conclusion was further supported by Coomassie blue gel-staining (Fig 5 lower) showing that ZnPP treatment did not alter the extracellular protein profiles. We also treated A2780 cells with 5 μM ZnPP for 72 hours and isolated exosomes from the medium. β-catenin was undetectable in the exosome proteins extracts (data not shown), further excluding the possibility that β-catenin protein is secreted in exosomes upon ZnPP treatment.

**Fig 6:**
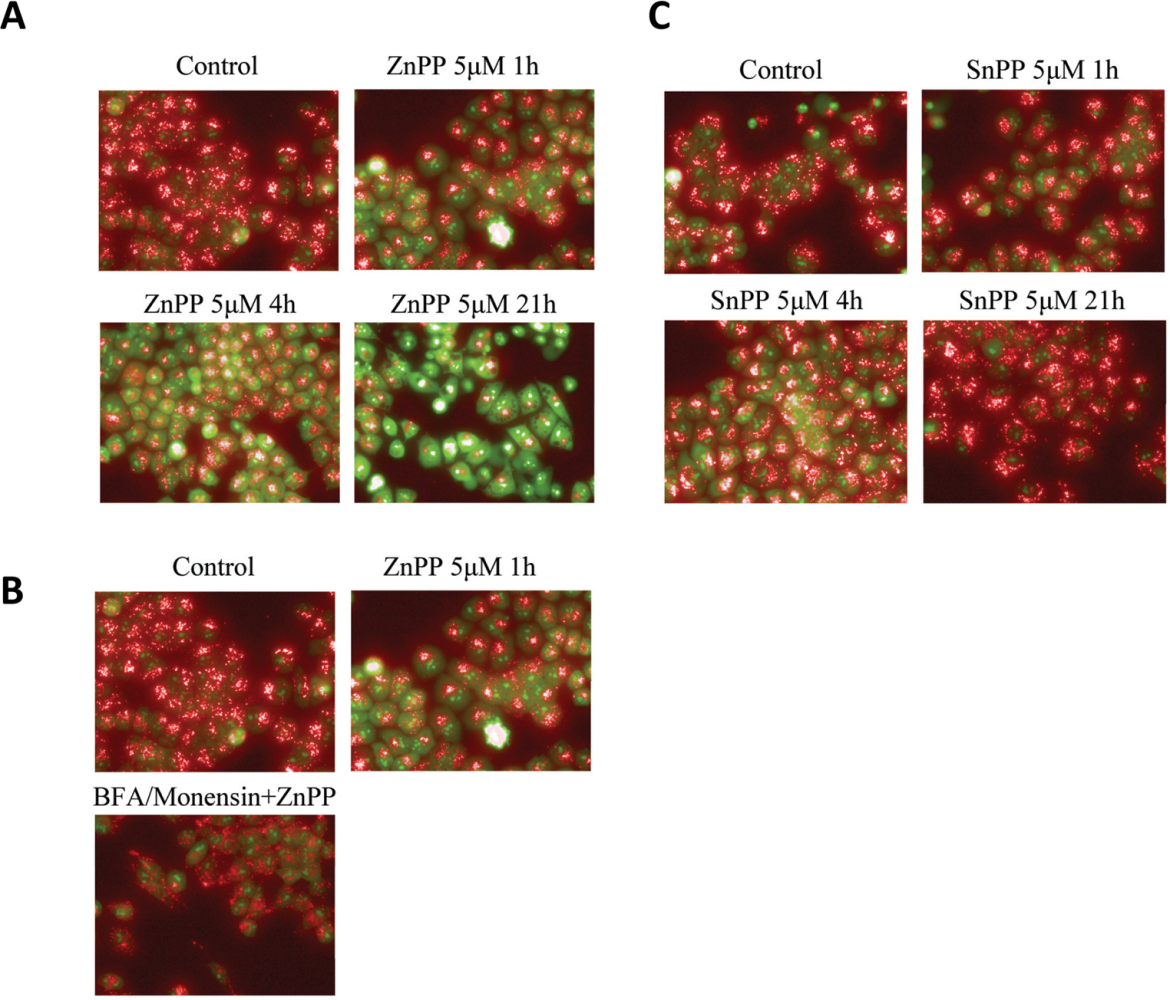
ZnPP enhances lysosome membrane permeability. A2780 cells were treated with 5 μM ZnPP (***A***) or 5 μM SnPP (***B***) for 1, 4 or 21 hours. Cells were then incubated with 2.5 μM AO for 30 minutes at 37°C. After incubation, cells were washed twice with HBSS. Images were captured using a fluorescent microscope (60X) with excitation at 500 nm, emission at 526 nm for AO green, excitation at 460 nm, emission at 650 nm for AO red. ***C*.** A2780 cells were pretreated with 21.2 μM Brefeldin A and 4 μM Monensin for 4 hours. The cells were then treated with 5 μM ZnPP for 1 hour followed by incubation with 2.5 μM AO for 30 minutes at 37°C. After incubation, cells were washed twice with HBSS. Images were captured using a fluorescent microscope (60X) with excitation at 500 nm, emission at 526 nm for AO green, and excitation at 460 nm, emission at 650 nm for AO red. Shown are representative images of three individual experiments.

The lysosome pathway is likely involved in ZnPP-induced β-catenin protein degradation. We have previously shown that lysosomal enzymes can be released upon enhanced lysosome membrane permeability, leading to cleavage of cellular proteins [14]. To determine whether the lysosome protein degradation pathway is involved in ZnPP-induced suppression of β-catenin protein expression, we examined lysosome membrane permeability after ZnPP treatment. AO was used to study the lysosome membrane permeability [14,24]. AO preferentially accumulates in the lysosomes and will emit red fluorescence when excited under acidic conditions. When the lysosome is permeabilized, AO will relocate to the cytosol where it emits a green fluorescence upon excitation. The shift from red to green fluorescence indicates an increase in lysosome membrane permeability [25]. As shown in Fig 6A, treatment with 5 μM ZnPP for 1, 4, or 21 hours induced a time-dependent shift in AO staining from red to green, indicating that ZnPP enhances lysosome membrane permeability in A2780 cells. In contrast, treatment with SnPP did not result in significant changes in membrane permeability (Fig 6C), consistent with our previous observation that SnPP doesn’t induce suppression of β-catenin protein expression.

**Fig 7:**
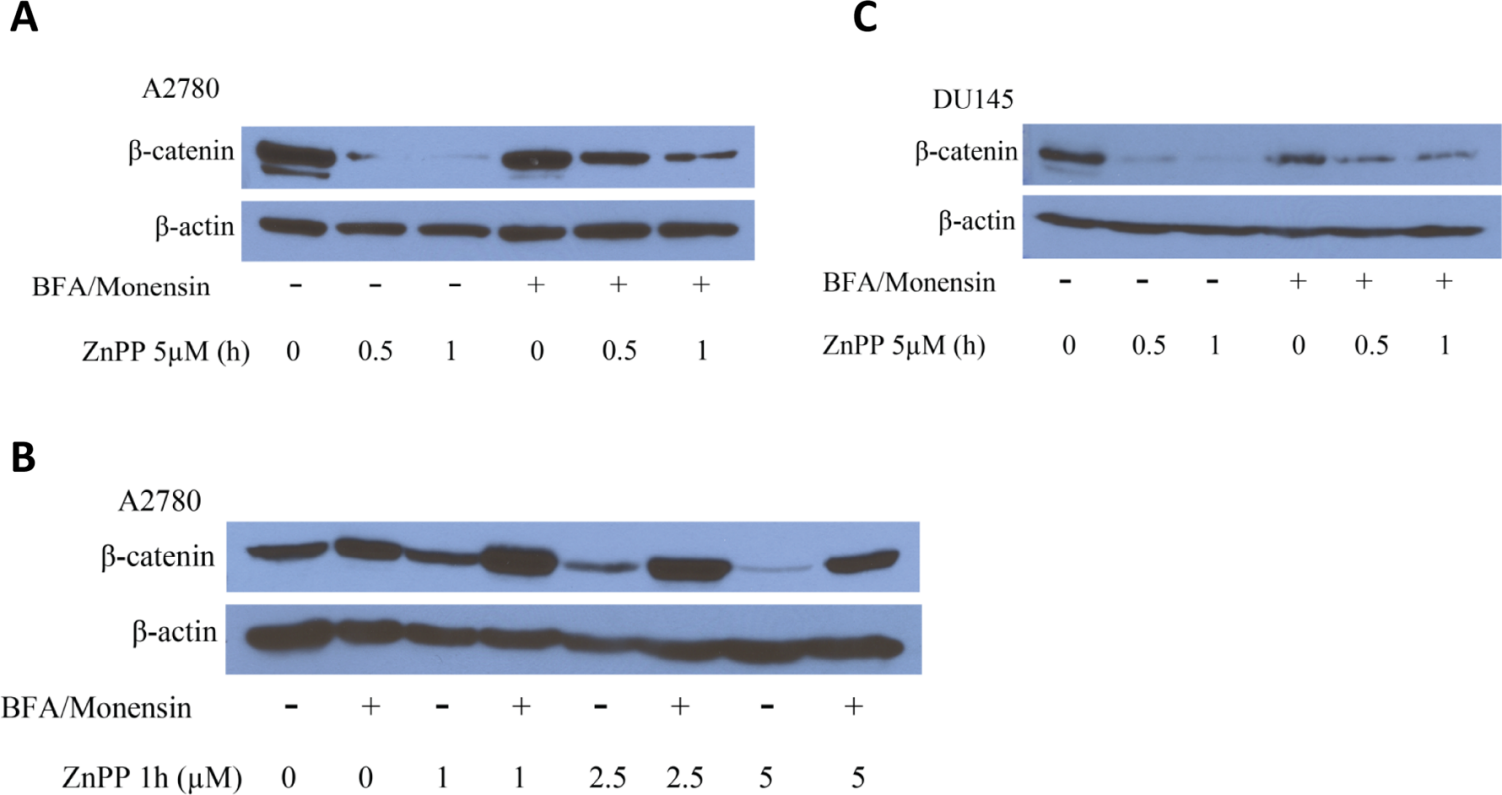
The BFA/Monensin cocktail attenuates ZnPP induced suppression of β-catenin expression. A2780 ***(A)*** or DU145 ***(B)*** cells were pretreated with or without 21.2 μM BFA and 4 μM Monensin overnight followed by treatment with 5 μM of ZnPP for 0.5 or 1 hour. ***C*.** A2780 cells were pretreated with 21.2 μM BFA and 4μM Monensin overnight followed by treatment with ZnPP for 1 hour at indicated concentrations. Cells were harvested and lysed. Western blot analysis was performed using antibodies against β-catenin and β-actin. Shown are representative images of three individual experiments.

BFA is known to block intracellular transportation of lysosomal enzymes [26] and Monensin can block acidification of lysosomes [27,28]. Therefore we tested whether a BFA/Monensin cocktail could reverse the lysosome membrane permeability induced by ZnPP. After pretreatment of the cells with a BFA/Monensin cocktail (final concentration of 21.2 μM BFA and 4 μM Monensin) overnight (Fig 6B), a reversal in the ZnPP-induced lysomal membrane permeability was observed. These results support the involvement of the lysosome degradation pathway in the ZnPP-induced suppression of β-catenin protein expression. To confirm this assumption, A2780 and DU145 cells were treated with the BFA/Monensin cocktail overnight and treated with ZnPP at the indicated concentrations and durations (Fig 7). ZnPP-induced suppression of β-catenin protein expression was significantly attenuated by pretreatment with the BFA/Monensin cocktail. The effect of ZnPP on β-catenin was both dramatic and significant and the reversal by BFA/Monensin was only observed after 0.5 and 1 hour ZnPP treatment (data not shown). However, the attenuation correlated with the concentration and duration of ZnPP treatment. These observations further demonstrate that the lysosome degradation pathway is likely involved in ZnPP-induced suppression of β-catenin protein expression.

## Discussion

The present study was designed to explore the potential cellular mechanisms that mediate ZnPP-induced suppression of β-catenin expression using cancer cell model systems. The most interesting finding from this study is that ZnPP-induced β-catenin protein degradation is accompanied by a significant inhibition of the ubiquitin-proteasome degradation pathway; and lysosome-mediated protein degradation seems to mediate this event. These results further elucidate the cellular mechanisms of ZnPP’s anticancer activity and indicate a potential new strategy in targeting the β-catenin Wnt signaling pathway for cancer therapy.

β-catenin protein levels are well controlled by phosphorylation and ubiquitin-proteasome degradation [9]. Therefore, we initially believed that ZnPP would activate the proteasome degradation pathway, thereby leading to rapid β-catenin protein degradation. However, several lines of experimental evidence indicated that proteasome degradation does not directly mediate ZnPP-induced β-catenin protein degradation. First, β-catenin protein phosphorylation, an event leading to the ubiquitination and subsequent proteasome degradation of β-catenin, was not induced by ZnPP in cancer cells. On the contrary, ZnPP treatment rapidly reduced phosphorylated β-catenin protein levels, likely due to the rapid degradation of the total cellular β-catenin protein. Second, western blot analysis of poly-ubiquitinated proteins showed that ZnPP induces accumulation of poly-ubiquitinated proteins, suggesting that ZnPP acts as a proteasome inhibitor rather than an activator. Third, co-IP with a β-catenin antibody and western blot analysis of K48-linkage specific poly-ubiquitinated proteins demonstrated that poly-ubiquitinated β-catenin accumulated after ZnPP treatment, consistent with its inhibition of proteasome activity. Lastly, a direct measurement of the proteasome chymotryptic, tryptic and caspase-like activities confirmed that ZnPP significantly suppresses proteasome activities in a time- and concentration-dependent manner in cancer cells. To our knowledge, this is the first demonstration that ZnPP is a proteasome inhibitor. Note that ZnPP’s proteasome inhibitory activity is different from the previously established proteasome inhibitors, such as MG132 [21,22], in that ZnPP seems to have a broader spectrum of proteasome inhibitory activity (Figs 3 and 4).

The possibility that ZnPP might induce β-catenin protein exportation through exosomes [23] thereby diminishing cellular β-catenin protein expression was also not supported by our experimental results. β-catenin protein was undetectable by western blot analysis of the extracellular proteins collected from the conditioned media of ZnPP-treated cells. Furthermore, the exosomes isolated from the media did not contain β-catenin proteins. These observations indicate that ZnPP does not induce β-catenin protein secretion from cancer cells.

We have recently reported that zinc ionophores enhance lysosome membrane permeability leading to the release of lysosomal enzymes and cleavage of cellular proteins [14]. In the present study, the use of AO allowed us to demonstrate that ZnPP enhances lysosome membrane permeability in our cancer cell model system, suggesting that ZnPP’s suppression β-catenin protein expression is a result of lysosomal enzyme digestion of cellular proteins. Importantly, ZnPP-induced lysosome membrane permeability could be effectively attenuated by pretreatment of the cells with the protein transportation inhibitory cocktail BFA/Monensin [29], which is known to block cellular transportation of lysosome enzymes (BFA, [26]) and inhibits lysosome acidification (Monensin, [27]). While this inhibitory cocktail is not specific to the lysosomes, the use of Monensin and BFA to alter lysosome structure and activity has been well documented [30–32]. These observations support the concept that the lysosome-mediated protein degradation pathway is involved in the ZnPP-induced suppression of β-catenin expression. Pretreatment of cancer cells with the BFA/Monensin cocktail significantly attenuated the suppression of β-catenin protein expression by ZnPP further confirming the involvement of the lysosomal protein degradation pathway in this process. It remains to be determined whether specific lysosomal enzymes are responsible for ZnPP-induced β-catenin protein degradation or whether a select group of proteins are degraded through this process in cancer cells. The potential interaction of the ubiquitin-proteasome system with the lysosome degradation pathway [33,34] that may account for ZnPP-induced β-catenin protein degradation is under active investigation. Given that there are no previous reports on lysosome-mediated suppression of β-catenin expression, the findings from the present study provide new insight into ZnPP’s anticancer activity and reveal potential new strategies in suppressing the canonical Wnt signaling pathway.

In summary, we have explored cellular mechanisms that mediate the ZnPP-induced suppression of β-catenin expression in cancer cells. Our results indicate that the lysosome degradation pathway is likely involved in ZnPP’s suppression of β-catenin expression and that this process is accompanied by an inhibition of proteasome activity. These findings provide a novel cellular mechanism of ZnPP’s anticancer activity and implicate a new strategy for targeting the canonical Wnt signaling pathway.

## References

[1] Rattan S, Chakder S (2000) Influence of heme oxygenase inhibitors on the basal tissue enzymatic activity and smooth muscle relaxation of internal anal sphincter. J Pharmacol Exp Ther 294: 1009–1016. 10945853

[2] Hirai K, Sasahira T, Ohmori H, Fujii K, Kuniyasu H (2007) Inhibition of heme oxygenase-1 by zinc protoporphyrin IX reduces tumor growth of LL/2 lung cancer in C57BL mice. Int J Cancer 120: 500–505. 17066448 10.1002/ijc.22287

[3] Nowis D, Bugajski M, Winiarska M, Bil J, Szokalska A, Salwa P, et al (2008) Zinc protoporphyrin IX, a heme oxygenase-1 inhibitor, demonstrates potent antitumor effects but is unable to potentiate antitumor effects of chemotherapeutics in mice. BMC Cancer 8: 197 10.1186/1471-2407-8-197 18620555 PMC2478682

[4] Fang J, Greish K, Qin H, Liao L, Nakamura H, Takeya M, et al (2012) HSP32 (HO-1) inhibitor, copoly(styrene-maleic acid)-zinc protoporphyrin IX, a water-soluble micelle as anticancer agent: In vitro and in vivo anticancer effect. Eur J Pharm Biopharm 81: 540–547. 10.1016/j.ejpb.2012.04.016 22576132

[5] La P, Fernando AP, Wang Z, Salahudeen A, Yang G, Lin Q, et al (2009) Zinc protoporphyrin regulates cyclin D1 expression independent of heme oxygenase inhibition. J Biol Chem 284: 36302–36311. 10.1074/jbc.M109.031641 19850937 PMC2794746

[6] Tanaka S, Akaike T, Fang J, Beppu T, Ogawa M, Tamura F, et al (2003) Antiapoptotic effect of haem oxygenase-1 induced by nitric oxide in experimental solid tumour. Br J Cancer 88: 902–909. 12644828 10.1038/sj.bjc.6600830 PMC2377071

[7] Wang S, Avery JE, Hannafon BN, Lind SE, Ding WQ (2013) Zinc protoporphyrin suppresses cancer cell viability through a heme oxygenase-1-independent mechanism: The involvement of the Wnt/beta-catenin signaling pathway. Biochem Pharmacol 85: 1611–1618. 10.1016/j.bcp.2013.03.011 23523860

[8] Polakis P (2012) Drugging Wnt signalling in cancer. Embo J 31: 2737–2746. 10.1038/emboj.2012.126 22617421 PMC3380214

[9] Stamos JL, Weis WI (2013) The beta-catenin destruction complex. Cold Spring Harb Perspect Biol 5: a007898 10.1101/cshperspect.a007898 23169527 PMC3579403

[10] Ciechanover A (2013) Intracellular protein degradation: from a vague idea through the lysosome and the ubiquitin-proteasome system and onto human diseases and drug targeting. Bioorg Med Chem 21: 3400–3410. 10.1016/j.bmc.2013.01.056 23485445

[11] Yang G, Nguyen X, Ou J, Rekulapelli P, Stevenson DK, Dennery PA (2001) Unique effects of zinc protoporphyrin on HO-1 induction and apoptosis. Blood 97: 1306–1313. 11222374 10.1182/blood.v97.5.1306

[12] Ding WQ, Liu B, Vaught JL, Yamauchi H, Lind SE (2005) Anticancer activity of the antibiotic clioquinol. Cancer Res 65: 3389–3395. 15833873 10.1158/0008-5472.CAN-04-3577

[13] Zhang X, Yu H, Lou JR, Zheng J, Zhu H, Popescu NI, et al (2011) MicroRNA-19 (miR-19) regulates tissue factor expression in breast cancer cells. J Biol Chem 286: 1429–1435. 10.1074/jbc.M110.146530 21059650 PMC3020751

[14] Yu H, Zhou Y, Lind SE, Ding WQ (2009) Clioquinol targets zinc to lysosomes in human cancer cells. Biochem J 417: 133–139. 10.1042/BJ20081421 18764784

[15] Zhang Z, Bi C, Schmitt SM, Fan Y, Dong L, Zuo J, et al (2012) 1,10-Phenanthroline promotes copper complexes into tumor cells and induces apoptosis by inhibiting the proteasome activity. J Biol Inorg Chem 17: 1257–1267. 10.1007/s00775-012-0940-x 23053530 PMC3662054

[16] Komander D (2009) The emerging complexity of protein ubiquitination. Biochem Soc Trans 37: 937–953. 10.1042/BST0370937 19754430

[17] Cvek B, Milacic V, Taraba J, Dou QP (2008) Ni(II), Cu(II), and Zn(II) diethyldithiocarbamate complexes show various activities against the proteasome in breast cancer cells. J Med Chem 51: 6256–6258. 10.1021/jm8007807 18816109 PMC2574941

[18] Kim I, Kim CH, Kim JH, Lee J, Choi JJ, Chen ZA, et al (2004) Pyrrolidine dithiocarbamate and zinc inhibit proteasome-dependent proteolysis. Exp Cell Res 298: 229–238. 15242777 10.1016/j.yexcr.2004.04.017

[19] Pan J, Zhang Q, Wang Y, You M (2010) 26S proteasome activity is down-regulated in lung cancer stem-like cells propagated in vitro. PLoS One 5: e13298 10.1371/journal.pone.0013298 20949018 PMC2952619

[20] Murata S, Yashiroda H, Tanaka K (2009) Molecular mechanisms of proteasome assembly. Nat Rev Mol Cell Biol 10: 104–115. 10.1038/nrm2630 19165213

[21] Lee DH, Goldberg AL (1998) Proteasome inhibitors: valuable new tools for cell biologists. Trends Cell Biol 8: 397–403. 9789328 10.1016/s0962-8924(98)01346-4

[22] Alexandrova A, Petrov L, Georgieva A, Kirkova M, Kukan M (2008) Effects of proteasome inhibitor, MG132, on proteasome activity and oxidative status of rat liver. Cell Biochem Funct 26: 392–398. 10.1002/cbf.1459 18236383

[23] Chairoungdua A, Smith DL, Pochard P, Hull M, Caplan MJ (2010) Exosome release of beta-catenin: a novel mechanism that antagonizes Wnt signaling. J Cell Biol 190: 1079–1091. 10.1083/jcb.201002049 20837771 PMC3101591

[24] Boya P, Kroemer G (2008) Lysosomal membrane permeabilization in cell death. Oncogene 27: 6434–6451. 10.1038/onc.2008.310 18955971

[25] Erdal H, Berndtsson M, Castro J, Brunk U, Shoshan MC, Linder S (2005) Induction of lysosomal membrane permeabilization by compounds that activate p53-independent apoptosis. Proc Natl Acad Sci U S A 102: 192–197. 15618392 10.1073/pnas.0408592102 PMC544072

[26] Oda K, Nishimura Y (1989) Brefeldin A inhibits the targeting of cathepsin D and cathepsin H to lysosomes in rat hepatocytes. Biochem Biophys Res Commun 163: 220–225. 2775262 10.1016/0006-291x(89)92124-4

[27] Misinzo G, Delputte PL, Nauwynck HJ (2008) Inhibition of endosome-lysosome system acidification enhances porcine circovirus 2 infection of porcine epithelial cells. J Virol 82: 1128–1135. 18032516 10.1128/JVI.01229-07 PMC2224462

[28] Pohlmann R, Kruger S, Hasilik A, von Figura K (1984) Effect of monensin on intracellular transport and receptor-mediated endocytosis of lysosomal enzymes. Biochem J 217: 649–658. 6231917 10.1042/bj2170649 PMC1153265

[29] O'Neil-Andersen NJ, Lawrence DA (2002) Differential modulation of surface and intracellular protein expression by T cells after stimulation in the presence of monensin or brefeldin A. Clin Diagn Lab Immunol 9: 243–250. 11874859 10.1128/CDLI.9.2.243-250.2001 PMC119934

[30] Lippincott-Schwartz J, Yuan L, Tipper C, Amherdt M, Orci L, Klausner RD, et al (1991) Brefeldin A's effects on endosomes, lysosomes, and the TGN suggest a general mechanism for regulating organelle structure and membrane traffic. Cell 67: 601–616. 1682055 10.1016/0092-8674(91)90534-6

[31] Chi C, Zhu H, Han M, Zhuang Y, Wu X, Xu T (2010) Disruption of lysosome function promotes tumor growth and metastasis in Drosophila. J Biol Chem 285: 21817–21823. 10.1074/jbc.M110.131714 20418542 PMC2898421

[32] Janda E, Nevolo M, Lehmann K, Downward J, Beug H, Grieco M (2006) Raf plus TGFbeta-dependent EMT is initiated by endocytosis and lysosomal degradation of E-cadherin. Oncogene 25: 7117–7130. 16751808 10.1038/sj.onc.1209701

[33] Raiborg C, Stenmark H (2009) The ESCRT machinery in endosomal sorting of ubiquitylated membrane proteins. Nature 458: 445–452. 10.1038/nature07961 19325624

[34] Rogov V, Dotsch V, Johansen T, Kirkin V (2014) Interactions between autophagy receptors and ubiquitin-like proteins form the molecular basis for selective autophagy. Mol Cell 53: 167–178. 10.1016/j.molcel.2013.12.014 24462201

